# Supplementary material for: Phenotypic and lifestyle determinants of HbA1c in the general population–The Hoorn Study
Source: PLoS One. 2020 Jun 4;15(6):e0233769. doi: 10.1371/journal.pone.0233769 (PMC7272077; doi:10.1371/journal.pone.0233769)
Supplement: Attachment — Submitted filename: Comment to Authors(PONE-D-19-25704).doc [file pone.0233769.s001.doc]

**Summary:**

This manuscript by Willem et al. entitled “Phenotypic and lifestyle deter**minants of HbA1c in the general population – the Hoorn Studies” is aimed at understanding the relevance of phenotypic and lifestyle factors to HbA1c, in the general population. The data of both cohorts, independent of FPG and 2hPG, higher age, female sex, larger waist circumference, and smoking were associated with a higher HbA1c level. Larger hip circumference, higher BMI, higher alcohol consumption and vitamin C intake were associated with a lower HbA1c level. FPG and 2hPG together explained 41.0% (HS) and 53.0% (NHS) of the total variance in HbA1c. The authors fully describe the relationship between lifestyle, blood glucose levels and glycated hemoglobin in NHS and HS. There are several concerns to consider.**

**Major:**

**This study is simply a description and fails to give a reasonable explanation. The results of BMI related to increased hip circumference and low-grade glycated hemoglobin are interesting, but there is no explanation. In general, glycated hemoglobin is a non-independent diagnostic standard for diabetes. In this study, as a key point to explore, the actual or predictive role of glycated hemoglobin changes in the model population should be more revealed. At present, I cannot appreciate the value of the information provided by the research to clinical or public nutrition research. I hope researchers can further discuss and reveal the significance of the research.**

**1．The trend of BMI results in HS from Q1 to Q4 is inconsistent. Can this result be adopted?**

**2. Hemoglobin and serum alanine aminotransferase are important factors affecting glycated hemoglobin, which are not shown in the analysis results, can they be explained?**

**3. Can I know the results about the investigator's medication history?**

**4. The author's study showed a negative correlation between the amount of alcohol consumed and the content of glycated hemoglobin, which means that increased drinking in healthy people is beneficial to prevent blood sugar rising.** **Can I understand it this way?**

**5. The author further explains the significance of the findings for metabolic or cardiovascular disease will be appreciated. (The significance of the study is unclear because the glycated hemoglobin in the study fluctuates within the normal range)**

**6. The conclusion that women's hemoglobin is higher than men's is also worthy of our attention. It would be greatly appreciated if the author could further analyze the pre- and post-menopausal women. (Because previous studies have shown that estrogen treatment can increase glycated hemoglobin levels)**
